# Editorial: Macroeconomic impact of disease dynamics: a temporal-spatial analysis

**DOI:** 10.3389/fpubh.2024.1473761

**Published:** 2024-09-03

**Authors:** John Yfantopoulos, Fanglei Zhong, Rasheda Khanam

**Affiliations:** ^1^Master in Business Administration Health Management National and Kapodistrian University of Athens, Athens, Greece; ^2^School of Economics, Minzu University of China, Beijing, China; ^3^Institute of Carbon Neutrality Development Research, Minzu University of China, Beijing, China; ^4^University of Southern Queensland, Toowoomba, QLD, Australia

**Keywords:** macroeconomics, epidemiology, public health, dynamics of diseases, spatial and temporal analysis

Improving health and longevity has always been at the forefront of the global health agenda of Governments and International Health Organizations across the universe.

The aim of this Research Topic is to stimulate the multidisciplinary dialogue to unravel the macroeconomic and epidemiological spatial and temporal dimensions on the determinants of health and its impact on health and quality of life. The post COVID-19 era has highlighted the need to integrate knowledge accumulated from the economic and health shocks and search for optimal, effective, efficient and equitable global health strategies. The COVID-19 pandemic generated an increasing interest among Public Health and Health Economic Researchers to implement longitudinal studies across different countries (e.g., Greece, Italy, the Netherlands, Russia, South Africa, Sweden, the United Kingdom, and the United States of America) (1). Several methodologies have been developed to assess the impact of COVID-19 on Health-Related Quality of Life (HRQOL, measured by EQ-5D-5L and EQ VAS), the mental wellbeing (WHO-5) the WellBeing Index, the Patient Health Questionnaire (PHQ)-9 and General Anxiety Disorder (GAD)-7 (2, 3).

**In Health Economics**, a voluminous literature has been studying the short- and long-term effects of micro and macroeconomic variables like income, health expenditure, unemployment, education, housing, access to health services, insurance coverage, tobacco and alcohol consumption, on health status across the universe. Often life expectancy, infant and child mortality as well as standardized for age and sex death rates have been used as proxies for the measurement of health status for different countries, regions and socio-economic groups (4).

Health economic historians have investigated the spatial and temporal dimensions of life expectancy from the prehistoric times to twenty first century. In prehistoric times, primitive sanitary and living conditions high rates of accidents and infectious diseases resulted to short human longevity of 20 years. Twentieth century advances in medical care, nutritious diet, reforms in health systems and technological improvements almost quadruple life expectancy. However, despite the continues improvements in longevity there are substantial geographical/spatial inequalities among the nations ranging from 52.5 years in Chad, Nigeria and Central Africa to 86 years in Monaco, Hong Kong, and Japan (5).

Health is an investment contributing to human capital, human productivity and long-term economic growth. Health economics used long term methodologies covering the course of 100–125 years to assess the impact of health on the growth paths of selected industrialized countries (6). Using aggregate time series pool data from OECD, Eurostat, UN and World Bank data sources for selected decades covering the period 1960–2022 several health economists attempted to analyze (i) the relationship and causality between health (using life expectancy as an indicative indicator) and income, health expenditure, education, occupation, alcohol consumption, tobacco, physical activity, and improvements in water and sanitation and (ii) to specify full information Maximum Likelihood Models and Distributed Lag Models to ascertain the dynamic/temporal and spatial effects of the above factors to improvements in health and quality of life (7, 8).

**Epidemiologists** have explored longitudinal studies to examine and monitor the impact of risk factors on health outcomes. Their research designs vary enormously in their objectives, sample sizes, and modeling, taking the form of observational or experimental specifications (9–11). Several studies have identified spatiotemporal patterns and risk factors associated with various infectious diseases. For instance, the spatiotemporal analysis of COVID-19 mortality in the U.S. revealed high-risk clusters and associations with social determinants of health, such as unemployment and residential segregation (12). Various methodological approaches have been employed to study the spatiotemporal epidemiology of diseases. Bayesian models have been widely used to incorporate spatial, temporal, and spatiotemporal effects, as seen in the studies on COVID-19 (13). The use of spatial autocorrelation and spatiotemporal statistics has been effective in identifying clusters and high-risk areas, as demonstrated in the studies on pertussis in China (14).

Some longitudinal studies like the British Office Population Study runs for decades. Health outcomes and incidence of cancer have been investigated in relation to employment status, housing, lifestyle, and other variables over successive waves of censuses. The Framingham Heart study runs since 1948 and it is recognized as “the quintessential longitudinal study in the history of medical research” (15).

A major problem of longitudinal studies, which is often recorded in NCD and chronic diseases such as diabetes, heart disease, and cancer is that large numbers of people must be followed for long periods to achieve statistically meaningful results. In the future better-quality controls in the collection of epidemiological information would improve the investigation of causal relationships between risk factors and patterns of diseases and reduce the bias in the data that may be subject to ecological fallacies.

Under this Research Topic, five articles have been successfully published, each offering valuable insights into various aspects of the disease-economy nexus.

Del Castillo et al. introduce a novel approach by utilizing electricity consumption (EC) and nighttime lights (NTL) datasets to estimate the economic losses from COVID-19 lockdowns in the Philippines. Their innovative method provides a rapid means of assessing economic impact, crucial for balancing health and economic concerns during pandemic emergencies. The study reveals that models based on EC were superior in explaining GDP compared to those using NTL, with a combination of both improving prediction accuracy.

Tavares employs fixed-effects panel data linear regression and quantile linear regression to identify factors influencing treatable mortality rates across European countries. The study uncovers gender-specific drivers of treatable mortality, with common factors such as GDP per capita and health expenditures having favorable effects, while nurses and beds per capita showed unfavorable impacts. These findings underscore the need for targeted healthcare policies and resource allocation strategies.

Zhou et al. focus on the economic burden of congenital heart disease in underdeveloped areas of China, using a multi-stage stratified cluster sampling method. Their analysis reveals increasing trends in average inpatient costs and daily inpatient costs from 2015 to 2020, while out-of-pocket expenses decreased. This study highlights the significant economic impact of congenital heart disease on families and society, emphasizing the need for targeted healthcare interventions in economically disadvantaged regions.

Silva et al. develop an innovative modeling framework based on Geographic Information Systems and spatial analysis to identify municipalities where population density strongly correlates with disease incidence and commuting patterns. Their approach, particularly useful for short-term predictions with limited data, provides a valuable tool for public health decision-making in resource-constrained settings.

Demsash utilizes the 2019 Ethiopian Mini Demographic and Health Survey dataset to examine the spatial distribution and factors influencing household enrollment in Community-Based Health Insurance (CBHI). The study reveals inadequate enrollment levels and identifies key factors such as educational status, age, wealth status, and media exposure as significant determinants of CBHI participation. This Research Topic highlights the importance of considering spatial heterogeneity in health insurance policy implementation.

In the future, health economists and epidemiologists, should account for unobserved heterogeneity, cross-section dependence and temporal dynamics by exploring more advanced panel econometric and epidemiological methods based on micro/individual data.

This Research Topic highlights the critical role of spatiotemporal analysis in understanding the macroeconomic and epidemiological dimensions of diseases and health outcomes. By identifying patterns, risk factors, and methodological approaches, these studies provide valuable insights for public health planning and targeted interventions. The integration of socioeconomic and demographic factors further enhances the understanding of disease dynamics, enabling more effective and equitable health strategies. Continued research and the application of advanced spatiotemporal methods are essential for addressing current and future public health challenges.
